# Subclinical neurological and balance abnormalities in late-onset auditory neuropathy spectrum disorder (ANSD) as a part of multisystem neurological presentation

**DOI:** 10.3934/Neuroscience.2026018

**Published:** 2026-07-23

**Authors:** Prashasti P Poovaiah, Atchayaram Nalini, Seena Vengalil, Suman Narayaswamy, Yamini Belur Keshavaprasad, Aravind Kumar Rajasekaran, Pradeep Yuvraj

**Affiliations:** 1 Department of Speech and Hearing, Father Muller College, Mangalore, Karnataka PO 575002, India; 2 Department of Neurology, National Institute of Mental Health and Neuroscience (NIMHANS), Bangalore, Karnataka PO 560029, India; 3 Department of Speech Pathology and Audiology, National Institute of Mental Health and Neuroscience (NIMHANS), Bangalore, Karnataka PO 560029, India

**Keywords:** auditory neuropathy, proprioception, nerve conduction, somatosensory evoked potential, vestibulopathy

## Abstract

Auditory neuropathy spectrum disorder (ANSD) is primarily considered a hearing disorder, with balance issues rarely reported. Although some studies have investigated vestibular abnormalities in ANSD in addition to auditory deficits, the neurological aspects have largely been overlooked. Subtle balance deficits in individuals with ANSD often go unnoticed, which has resulted in limited research in this area. The present study aims to describe a comprehensive battery of neurological and audio-vestibular tests in individuals with acquired ANSD who reported balance-related complaints but did not have a prior diagnosis of a neurological disorder, thus representing clinically under-recognized deficit. Fourteen patients with ANSD underwent a series of clinical assessments, including auditory brainstem response (ABR), cervical and ocular vestibular evoked myogenic potentials (VEMPs), the video head impulse test (vHIT), motor and sensory nerve conduction studies, somatosensory evoked potentials (SSEP), hematological investigations, and magnetic resonance imaging (MRI). All patients were Romberg-positive and showed an impaired tandem gait, despite largely preserved cranial nerves and motor examination findings on routine clinical testing. Auditory assessments revealed varying degrees of hearing loss, absent ABRs, and abnormal VEMPs and vHIT results, while MRI findings were unremarkable. Motor nerve conduction was normal in most patients; however, a few individuals exhibited reduced or absent sensory nerve potentials. In addition, SSEP latencies were abnormal in nine patients. The absence of VEMPs and abnormal vHIT findings suggest an underlying vestibulopathy, whereas nerve conduction and SSEP abnormalities indicate a possible somatosensory deficit in ANSD. These preliminary findings underscore the importance of a multidisciplinary approach to the evaluation of balance deficits in ANSD, an area in which evidence remains scarce.

## 1. Introduction

Recently, that first reports of patients with ‘hearing problems’ with normal audiometric thresholds were reported in the literature [1]–[3]. The earlier term coined for this disorder was ‘auditory neuropathy’ [4], considering the fact that the sensory organs in the cochlea, namely the outer hair cells, showed normal functioning, and the auditory nerve was affected by a neuropathic disorder. This disorder severely impairs the perception of regular conversational speech, causing individuals to be communicatively deficient. The terminology was changed to ‘Auditory Neuropathy Spectrum Disorder’ (ANSD) [5] to address the potential confusion that arises from multiple designations.

ANSD has been widely reported across diverse populations worldwide, with prevalence estimates varying depending on the screening protocols. Studies from North America have reported ANSD in approximately 0.5%–1% of infants [6]. The incidence of ANSD was reported to be 5.3% out of the 487 children tested [7] in an Indian study, whereas a large group study reported from United Kingdom reported it to be 0.39/1000 births [8]. The incidence of acquired or late-onset ANSD was reported to be 1.78% of 1343 adults with sensorineural hearing loss [9]. When ANSD occurs in adults as an acquired condition (late-onset), it is characterized by mild-to-severe fluctuating hearing loss [4],[10] and poor speech discrimination in quiet regions [11]. Auditory brainstem responses (ABR) are abnormal or absent, thus indicating dyssynchronous neural firing, accompanied by the presence of otoacoustic emissions (OAE) [4],[10], thus indicating normal cochlear outer hair cell functioning.

Individuals with ANSD demonstrated a notable prevalence of symptoms such as imbalance and dizziness [12]. Video head impulse test (vHIT) recording has revealed the presence of overt and covert saccades [13], and abnormal and absent responses on cervical vestibular evoked myogenic potentials (cVEMP)and ocular vestibular evoked myogenic potentials (oVEMPs) indicate widespread involvement of both the superior and inferior divisions of the vestibulo-cochlear nerve [14],[15]. Advanced techniques such as galvanic vestibular stimulation (GVS)-VEMP demonstrated preserved neural responses despite the absence of acoustic VEMP, thereby localizing the lesion to the retro-cochlear site [16]. In addition, abnormality was reported in a caloric test [17].

The pathophysiology of ANSD is attributed to presynaptic (involving outer or inner hair cells), synaptic, and postsynaptic lesions in the auditory pathway [18], although it is difficult to distinguish the anatomical location. Genetic studies have identified the OTOF, DFNB9, MPZ, GJB2, PMP22, and OPA1 genes as a few non-syndromic causative factors for ANSD [19]. Several neurological conditions present with ANSD, including Friedreich's ataxia, Charcot-Marie-tooth disease (CMT), brainstem demyelinating conditions [20], Brown-Vialetto-Van Laere syndrome (BVVL) [21], Madras Motor Neuron Disease (MMND) [22], autosomal dominant or recessive optic atrophy [23], and myelin disorders [4].

The term ANSD was coined to encompass a spectrum of disorders that affect various sites of lesions ‘other than the auditory nerve’ [5]. Although Hayes and Sininger (2008) did not specify these sites, it can be assumed that they were referring to the auditory inner hair cells, ribbon synapse, and auditory axons, or lesions beyond auditory pathways.

Individuals with late-onset ANSD report marked difficulties in speech comprehension, in quiet, in noisy surroundings, or in both, which forms the majority of the research [4]. The literature reveals numerous neurological characteristics associated with this condition [24],[25]. Individuals with late-onset ANSD report difficulty in maintaining balance and the fear of falling, which may indicate vestibular neuropathy [26]. These individuals may be varying vestibular symptoms can be asymptomatic, and may or may not have concomitant peripheral neuropathies [26],[27]. The literature sparsely reports subclinical neurological and balance abnormalities in individuals with ANSD. Although there are reports of vestibular evaluations performed in individuals with ANSD, through questionnaires [12], VEMPs [28]–[30], and the vHIT [13], these studies do not report neurological test findings among their participants.

Patients presenting with hearing difficulties as their primary complaint are frequently and predominantly evaluated from an auditory perspective, with limited assessments of potential motor, sensory, and balance system deficits. This study seeks to address this gap by investigating evidence for subclinical neurological and balance abnormalities in ANSD patients. Through comprehensive audio-vestibular and neurological evaluations, we seek to characterize the broader neurological involvement associated with ANSD and provide evidence that supports a more integrated and holistic framework for its diagnosis and clinical management.

## 2. Methods

This study employed a prospective observational design based on a clinical cohort. The patients (n = 14) included in the study were evaluated at a tertiary care neuropsychiatric hospital. All of them presented to the outpatient unit of the Department of Speech Pathology and Audiology with a primary complaint of difficulty in comprehending speech, with hearing loss acquired in the second decade of life. Audiological and associated symptoms were assessed and documented as part of the neuro-audiological case history. Additionally, they reported balance-related difficulties in daily activities, which led to further evaluations at the Department of Neurology. Audiological and neurological assessments were conducted as part of a routine clinical protocol and served as pre-inclusion criteria for a larger research study that examined speech perception in individuals with ANSD.

The study was approved by the Institute Ethics Committee of National Institute of Mental Health and Neuroscience (NIMHANS), and we confirm adherence to the guidelines set by the Declaration of Helsinki. Informed written consent was obtained from all adult participants, and assent was obtained from the guardians in the case of minors.

### 2.1. Audiological test

The diagnosis of ANSD was done based on a standard protocol [4]. The audiological tests included pure-tone audiometry (PTA), as well as speech audiometry consisting of speech recognition threshold (SRT), speech recognition scores (SRS), speech awareness level (SAL), and speech in noise test (SPIN) (Interacoustics AC-40: Interacoustics, Denmark). PTA was administered between 0.25 kHz and 8 kHz for air conduction and between 0.25 kHz and 4 kHz for bone conduction measurements using the modified Hughson and Westlake procedure (ANSI S3.21–1978 (R-1997) [31]. Speech audiometry was administered using monitored live-voice presentation, wherein the examiner delivered using a standardized word list through a microphone routed via the audiometer. The presentation level was kept typically at a 30–40 dB sensation level and the output levels were continuously monitored using the volume-unit meter of the audiometer. Tympanometry and acoustic stapedial reflexes (GSI-tympstar Pro: Grason StadlerInc., MN) were performed, and the tympanograms were graded as per Jerger's method of classification [32].

### 2.2. Oto-acoustic emission

Distortion product otoacoustic emissions (DPOAE) (ILO 292 USB II hardware and ILO v6 software; Otodynamics Ltd., London) were recorded between 1 kHz and 6 kHz with the stimuli presented at 65/55 dB SPL.

### 2.3. ABR tests

ABRs were recorded on an IHS smart EP (Intelligent Hearing Systems Inc., Miami, FL) with the patient seated comfortably in a reclining chair. The electrode montage of vertex (CZ), the ipsilateral (stimulated) ear lobe, and the ground to the forehead (Fz) were measured. Click stimuli of 100 µs were used in rarefaction and condensation polarity at an intensity of 90dBnHL. The amplitude bandpass was set between 30–3000 Hz. The electrode impedances were roughly equivalent and were kept below <5 kΩ.

### 2.4. Vestibular test

The cVEMP and oVEMP were separately recorded using the IHS Smart EP (Intelligent Hearing Systems Inc., Miami, FL). For these tests, the electrode sites were prepared using an abrasive skin gel, and electrodes were placed with conductive paste and secured with skin tape. Standard protocols were followed to record the cVEMP and oVEMP [33]. Two consecutive recordings were obtained to ensure waveform reproducibility. The cVEMP and oVEMP results were compared with age-matched clinical norms. Additionally, the gains of the semicircular canals were measured using the vHIT (Natus Medical Inc., Middleton, USA) following standard procedures [34]. The stimulus and recording parameters for the ABR, cVEMP, and oVEMP are detailed in Table 1.

### 2.5. Neurological examination

The neurological evaluation consisted of examinations of the cranial nerves, motor system (tone and power), sensory system (superficial sensation, joint-position sense, timed vibration test, touch-pain-temperature sensation), cerebellar tests (finger-nose test, knee-heel test, and dysdiadochokinesis), deep tendon reflexes (DTR), and gait assessment.

### 2.6. Laboratory investigation

Patients underwent laboratory investigations, including the complete blood count (CBC), hemoglobin (Hb), and lipid profile. Tests for inflammation and autoimmunity included the erythrocyte sedimentation rate (ESR) and antinuclear antibodies (ANA). Tests for infection included serum serology for hepatitis B, hepatitis C, and HIV. A comprehensive metabolic panel consisting of the serum lactate level, ammonia (NH3), serum calcium level, serum creatine phosphokinase (CK), serum phosphate, homocysteine, vitamin B12, and folate level were evaluated.

**Table 1.** Stimulus and recording parameters for ABR, cVEMP, and oVEMP.

Parameters	ABR	cVEMP	oVEMP
Stimulus			
Transducer	Etymotic Research ER-3C insert earphones
Type	Click: 100 µs	Tone burst: 500 Hz; 1–0–1 cycle	Tone burst: 500 Hz; 1–0–1 cycle
Rate	11.1/s	4.1/s	4.1/s
Intensity	90 dBnHL	106 dBnHL	106 dBnHL
Polarity	Rarefaction	Rarefaction	Rarefaction
Presentation	Monoaural Ipsilateral	Monoaural Ipsilateral	Monoaural Contralateral
Recording			
Montage			
Positive	Vertex	The upper half of SCM	1cm below the center of the lower eyelid close to the inferior oblique in a vertical orientation.
Negative	Ipsilateral ear lobe	The inspectional base of SCM at Sternoclavicular junction	Below a positive electrode at about 1.5 cm on the cheek.
Ground	Contralateral ear lobe	Forehead	Forehead
Filter	100–3000 Hz	10–1500 Hz	1–1000 Hz
Amplification	100,000	5000	5000
Rejection Voltage			
EEG	30 µV	Open Window (100 µV)	Open Window (100 µV)
EMG	Not applicable	50–200 µV	1–100 µV
Post stimulus	12.8 ms	60ms	60ms
Pre-stimulus	−13 ms	−20 ms	−20 ms
Sweeps	1024	100	100
Trials	2	2	2

Note: ABR: auditory brainstem response; cVEMP: cervical vestibular evoked myogenic potential; oVEMP: ocular vestibular evoked myogenic potential; SCM: sternocleidomastoid; EEG: electroencephalogram; EMG: electromyogram; µs: microsecond; µV: microvolt; ms: millisecond.

### 2.7. Magnetic Resonance Imaging (MRI)

Brain magnetic resonance imaging (MRI) was performed on all participants using a standardized 3-Tesla protocol, including T1 and T2 weighted sequences, to assess for central nervous system abnormalities, specifically the cerebral cortex, subcortical white matter, brainstem, and cerebellum. The scans were reviewed by a neuroradiologist.

### 2.8. Nerve conduction and somatosensory test

The motor and sensory systems were objectively assessed using electrodiagnostics (EDX). The EDX evaluation consisted of motor conduction studies (MCS), sensory conduction studies (SCS), and somatosensory evoked potentials (SSEP) recorded using surface disk electrodes (Neuropack X1 MEB-9200K, Nihon Kohdencorp., Tokyo, Japan). MCS and SCS were performed for motor and sensory nerves of the upper and lower limbs. The motor nerves sampled were the median, ulnar, peroneal, and tibial nerves. The sensory nerves tested included the median, ulnar, sural, and superficial peroneal nerves (SPN). MCS and SCS measurements consisted of the compound muscle action potential (CMAP) and the sensory nerve action potential (SNAP). The latency, velocity, and amplitude of CMAP and SNAP were measured. Normative values for MCS and SCS were taken from the literature [35]. SSEP peak latencies were recorded from upper limbs (negative peak at 20 ms; N20) and lower limbs (positive and negative peak at 37 ms and 45 ms; P37-N45, respectively).

## 3. Results

### 3.1. History

The study consisted of 14 patients (8 females, 6 males), with a mean age of 18.8 years (±3.39). The mean age of onset of symptoms was 14.89 years (±1.41). The primary complaint of the patients was the total or partial inability to understand speech, which was reported as stationary or progressive. All the patients reported weakness in their lower limbs, which manifested as the difficulty to grip footwear, fatigue, or weakness in fingers on writing. All the subjects reported fatigue in carrying out physical activities. Additionally, they reported varying difficulties in sustaining balance while walking on uneven surfaces, climbing up and down stairs, and navigating in low-lit areas. The patients reported a ‘fear of fall’, with no episodes of actual falls. The patients had no familial history of hearing loss or neurological disease. The case history, audiological, neurological, MRI, & EDX reports are summarized in Table 2, with detailed data provided in the supplementary material.

**Table 2.** Summary of clinical, neurological, and electrophysiological findings in patients with ANSD (n = 14)

Domain	Parameter	Findings
Demographics	Age of onset	13–18 years
	Disease course	Static: 7/14; Progressive: 7/14
Clinical Symptoms	Hearing difficulty	Present in all patients (14/14)
	Balance complaints	Present in 6/14; absent in 8/14
	Fatigue	Present in 10/14; absent in 4/14
Cranial Nerve Findings	Extraocular movements, ptosis, diplopia, nystagmus	Normal in all patients (14/14)
	Facial involvement	Present in 2/14
	Tongue atrophy	Present in 3/14
	Tongue fasciculations	Present in 12/14
Motor System	Tone	Normal in 13/14; reduced in 1/14
	Power	Normal in all patients (14/14)
Cerebellar Function	Finger–nose, heel–knee, dysdiadochokinesia	Normal in all patients (14/14)
Sensory System	Superficial sensation	Impaired in 1/14
	Joint position sense	Impaired in 3/14
	Vibration sense	Reduced in 6/14
	Plantar response	Reduced in all patients (14/14)
Gait & Balance	Gait abnormality	Present in 4/14
	Romberg's test	Positive in all patients (14/14)
	Tandem gait	Impaired in all patients (14/14)
Clinical Impression	No definitive diagnosis (NDD)	5/14
	Suspected Brown-Vialetto-Van Laere (BVVL) syndrome	5/14
	Suspected riboflavin transporter deficiency	3/14
	Suspected axonal neuropathy	1/14

### 3.2. Clinical examination

#### 3.2.1. Cranial nerves

Clinical examinations of the cranial nerve functions revealed normal functioning of extra-ocular muscles, no ptosis, no spontaneous or gaze-evoked nystagmus, and no diplopia. Unilateral facial weakness was seen in 2 patients. Tongue fasciculations were observed in all patients. Tongue atrophy was seen in 2 patients, but none had tongue movement restrictions.

#### 3.2.2. Motor and sensory system

The motor system examination was normal except for patient 8, who had distal hypotonia in both the upper and lower limbs. Mild weakness of the thenar and hypothenar muscles, along with hammer toes, were seen in a patient. The sensory system evaluation showed a graded loss in lower limbs in a few and was normal in the rest of the subjects. Touch, pain, and temperature sensation were normal in all patients. The joint and position sense were either impaired or reduced in the lower limbs of 3 out of 10 patients tested. The timed vibration test was reduced in 6 out of 7 patients tested. The plantar response was flexor in all patients. The DTR was graded as either brisk or normal in all patients. The gait assessment showed mild waddling in 4 patients and a wide-based gait in 1 patient with no change in strides. Romberg's test and the tandem gait were positive in all patients. No deficits were found on the Cerebellar assessment.

#### 3.2.3. Laboratory investigation

Laboratory tests were performed as a part of the routine investigation done for peripheral neuropathy. Routine blood investigations such as CBC, Hb, ESR, electrolytes, TFT, and lipid profiles were within normal limits. The serum vitamin B12 values ranged between 74 pg/ml and 182.00 pg/ml in 5 patients and were within the normative range (197–771 pg/ml) for the rest of the patients. The serum homocysteine levels ranged between 22.8 µmol/L and 64.9 µmol/L among 5 patients (normative: 5–15 µmol/L). The serum lactate (normative: 4.5–19.8 mg/dL), NH₃ (normative: 15–45 µg/dL), serum calcium (normative: 8.6–10.2 mg/dL), serum creatine phosphokinase (CK) (normative: 20–200 U/L), and serum phosphate (normative: 2.5–4.5 mg/dL) levels were normal in all patients except patient 2, who had elevated serum lactate levels of 30 mg/dL. The serum serology, including hepatitis B, hepatitis C, and HIV, were negative for all patients.

#### 3.2.4. Audiological findings

The audiological test findings are summarized in Table 3 (details are provided as supplementary file). The audiological evaluation revealed PTA thresholds between 5–15 dB HL bilaterally in 3 patients, between 30–51.6 dB HL bilaterally among 6 patients, and between 51.6–76.6 dB HL bilaterally among 5 patients. SAL was established in all patients, as patients could not comprehend the speech stimuli. The tympanomtery was ‘A’ type along with an absence of acoustic stapedial reflexes bilaterally. ABRs were absent bilaterally in all subjects with the presence of a cochlear microphonic. The DPOAEs were present in all patients. The cervical and ocular VEMP were absent among 12 out of 14 patients. One patient showed bilateral presence of oVEMP, while the other demonstrated bilateral presence of both cVEMP and oVEMP. vHIT recordings revealed reduced gains in all three semicircular canals, along with the presence of overt and covert saccades in all patients.

#### 3.2.5. EDX measurements

EDX included MCS and SCS measures along with SSEP. The results are summarized in Table 3 and presented in detail in the supplementary material. MCS showed a reduced CMAP amplitude (1.4 mV) in the common peroneal nerve (CPN) in patient 3 (normative: ≥2–4 mV) and normal values in the rest of the subjects. A SNAP amplitude of 1.2 µV was recorded in the sural nerve in patient 6 (normative: ≥6 µV); an absent SNAP was recorded in sural and SPN in 7 patients.

SSEPs were recorded for the upper and lower limbs. The N20 recorded in the upper limbs ranged between 17.80–19.90 ms in all patients except in patient 2, which was 22.25 ms (normative: ~18–22 ms). The lower limb SSEP revealed a P37 (normative: ~37–45 ms) value between 37.60–42.30 ms in 6 patients and 35.00 ms in patients 3 and 11. The N45 ranged between 47.30–50.00 ms in 6 patients, whereas it was 42.60–44.30 ms in patients 3 and 11 (normative: ~45–55 ms). The MRIs were unremarkable in all subjects.

**Table 3.** Summary of the Electro-diagnostics and audiological findings in subjects with auditory neuropathy spectrum disorder (ANSD).

Domain	Parameter	Findings
Motor Conduction (MCS)	CMAP	Normal in 13/14; reduced in common peroneal nerve in 1/14
Sensory Conduction (SCS)	SNAP	Absent in sural ± superficial peroneal nerve in 7/14; absent in multiple nerves (ulnar, radial, sural) in 1/14; reduced in sural nerve in 1/14; normal in 6/14.
SSEP	Upper limb SSEP (N20)	Within normal limits in 13/14; prolonged latency in 1/14
	Lower limb SSEP (P37/N45)	Prolonged latencies in 5/14; absent responses in 4/14; normal in 5/14
Auditory Electrophysiology	ABR	Absent in all patients (14/14)
	Cochlear microphonics (CM)	Present in all patients (14/14)
	DPOAE	Present in all patients (14/14)
Vestibular Function	cVEMP	Absent in 12/14; present in 2/14
	oVEMP	Absent in 10/14; present in 4/14
	vHIT	Reduced gain/covert saccades in 12/14; normal in 2/14

Note: MCS: motor conduction study, SCS: sensory conduction study, SSEP: somatosensory evoked potential, CMAP: compound muscle action potential, SNAP: sensory nerve action potential, PTA: pure-tone average, SAL: speech awareness level, SRT: speech recognition threshold, SRS: speech recognition scores, SPIN: speech in noise test, ABR: auditory brainstem response, CM: cochlear microphonic, DPOAE: distortion product oto-acoustic emission, cVEMP: cervical vestibular evoked myogenic potential, oVEMP: ocular vestibular evoked myogenic potential, vHIT: video Head impulse test.

## 4. Discussion

Late-onset ANSD typically manifests during the second decade of life, with difficulty in speech comprehension being the predominant symptom. The diagnostic hallmark for ANSD include the absence of the ABR and the presence of OAEs, thus reflecting a loss of synchrony in the auditory nerve. While auditory deficits are predominant, these individuals may experience balance related symptoms that are often subtle, overlooked, or remain unreported.

### 4.1. Heterogeneity in audiological findings

The major complaint in our patients was difficulty in understanding speech, although the severity considerably varied, thus indicating heterogeneity of the disorder. The PTA thresholds in ANSD range from normal to profound [36], accompanied by the absence of an acoustic stapedial reflex, absent ABRs, and the presence of OAE's [36]. The findings of this study are consistent with this spectrum, thereby reflecting variability rather than reiterating a specific threshold distribution. All our patients had disproportionate SRT (or SAL) in relation to the PTA thresholds, thus indicating poor speech perception abilities, which is consistent with literature that show poor speech perception in ANSD [36],[37]. Furthermore, our patients had an absence of ABRs, with the presence of cochlear microphonic and DPOAEs indicating functional cochlear outer hair cells, which is a classic finding in ANSD [4],[36].

### 4.2. Balance deficits prompting multisystem evaluation

Although most of the patients reported difficulties maintaining balance while walking, a clinical examination of the motor system largely showed normal findings. The dissociation between the subjective imbalance and preserved motor system suggests involvements beyond primary motor pathways, potentially implicating multisensory involvement. The imbalance may arise due to deficits in the sensory system, the vestibular system, or the cerebellar system. Hence, the vestibular function was evaluated with cVEMP, oVEMP, and vHIT. The high prevalence of absent VEMPs suggests significant involvement of the otolith mediated vestibular pathways, which is consistent with previously reported literature [14]. Additionally, the vHIT results showed covert saccades and reduced gains, thus indicating dysfunction of all three semicircular canals. The results of abnormal VEMPs and vHIT findings indicate the presence of a bilateral vestibulopathy (BVP) [38],[39], though not necessarily attributed solely to ANSD pathology. Symptoms of BVP include movement-dependent postural dizziness and unsteadiness of stance and gait, which is aggravated in the dark and when walking on uneven surfaces [39],[40]. Notably, these hallmark symptoms closely mirrored the complaints described by the patients under study who reported difficulties walking on stairs, uneven surfaces, and poorly illuminated environments.

Beyond vestibular deficits, the clinical examination of the sensory system indicated a reduced timed vibration test, impaired joint-position sense in lower limbs, and a reduced Plantar flexor response. These findings may raise the possibility of a distal sensory neuropathy (DSP) [41],[42]. A blood investigation showed reduced vitamin B12 levels and increased homocysteine (in 6 of 14 patients), which is a biochemical abnormality associated with an idiopathic sensory neuropathy [43]. These findings further support the possibility of a co-existing systemic neurological process rather than a disorder confined to the auditory pathway.

All the patients were positive for Romberg's sign and an impaired tandem gait (on eyes open), along with a plantar flexor weakness. While a positive Romberg's sign is classically suggestive of dorsal column pathway dysfunction (proprioceptive sensory loss) [44], it may also indicate dysregulation in the vestibular or cerebellar pathways [44]. An abnormal tandem gait is generally associated with lesions of the cerebellar vermis and paraflocculus [45], and gait ataxia is associated with cerebellar pathology or a loss of proprioceptive inputs [46]. However, the targeted cerebellar examination revealed no deficits, and the brain MRIs were unremarkable in all our patients, thus effectively excluding demyelination, space-occupying lesions, and atrophy. These findings necessitated a further exploration of peripheral and proximal sensory pathways using an EDX evaluation.

### 4.3. Electrodiagnostic evidence of sensory pathway involvement

The amplitude of the CMAP measures the neuronal firing of the total number of motor nerve axons, and the amplitude of the SNAP is related to the neuronal firing of the sensory nerve axons. The SNAP were absent in the sural nerve and superior peroneal nerve in 7 patients, and a reduced amplitude was recorded in the sural nerve in 1 patient. In contrast, 6 patients demonstrated a normal SNAP. However, all 13 patients had normal CMAP, and only patient 1 had reduced CMAP in the CPN. The overall pattern reflects a selective sensory pathway involvement with relative sparing of motor fibers. Lesions which cause axon loss generally result in reduced CMAP and SNAP amplitudes. The pattern observed, absent SNAP with intact CMAPs, in the lower limbs raises a possibility of demyelination that affects the sensory fibers [47]. Alternatively, postganglionic lesions which involve the dorsal root ganglia (DRG) may account for absent SNAPs in lower limbs [48]. To further evaluate the sensory pathways, SSEPs were performed.

The proximal segments of the sensory peripheral nervous system can only be assessed by SSEPs, which study the sensory fibers from a distal point of stimulation to the spinal cord and somatosensory cortex. In this study, SSEPs recorded from the lower limbs showed prolonged latencies in 5 patients and absence in 4 patients despite having normal CMAP recordings. This reinforces the involvement of the proximal sensory pathway and supports a broader neurophysiological dysfunction. The utility of SSEPs to detect proximal demyelinating lesions in the presence of a normal distal CMAP has been documented in Guillain–Barré syndrome [49].

Pure proprioceptive loss without motor impairment is a rare presentation but has been reported in acquired neuropathies that involve the DRG [50],[51] and the central fibers proximally, particularly in the dorsal columns [49]. While the DRG loss can occur in some hereditary neuropathies, these conditions are often associated with other motor or cerebellar abnormalities [51]. However, none of the patients indicated cerebellar symptoms or atrophy. Interestingly, 4 patients with absent SSEPs in lower limbs also exhibited absent or reduced SNAP in the sural and superior peroneal nerves, thus reinforcing the hypothesis of dysfunction along the sensory afferent pathway.

An additional observation of interest was that patients with absent SNAP in the lower limbs or prolonged SSEPs also exhibited reduced vitamin B12 and increased homocysteine. Vitamin B12 deficiency is well known to cause peripheral neuropathy and posterior column dysfunction [52],[53], and SSEP abnormalities in this context have been previously documented [50]. These findings suggest that the vitamin B12 deficiency observed in 5/13 patients may contribute to the sensory pathway involvement. However, vitamin B12 deficiency alone cannot account for the full clinical picture.

Previous studies report abnormal ABRs in patients with a vitamin B12 deficiency [54], whereas all patients in the present study demonstrated absent ABRs irrespective of the vitamin B12 status.

Moreover, patients with normal vitamin B12 levels and normal SSEPs exhibited abnormal ABRs. Taken together, these findings suggest a complex clinical entity, where ANSD may represent one manifestation within a broader multisystem or neuro-degenerative process in at least a subset of patients.

### 4.4. Integrating vestibular and somatosensory dysfunction

Postural stability depends on the seamless integration of vestibular, visual, and somatosensory inputs. The vestibular system integrates visual inputs through the vestibulo-ocular-reflex pathway [55] and somatosensory inputs through dorsal-root axons and second-order neurons [55],[56]. Cortical and cerebellar regions that receive somatosensory inputs also project to vestibular nuclei [57]. Integrating vestibular and somatosensory information into the vestibular cerebellum is essential for posture control and perceiving self-motion [57],[58].

In this study, the patients exhibited concurrent vestibular and somatosensory deficits, as evidenced by reduced/absent SNAP, prolonged SSEP, and absent VEMPs. Rather than viewing these findings in isolation, they likely reflect the disruption of integrated sensory networks contributing to balance control. These findings raise a possibility of disrupted afferent inputs reaching the vestibular nuclei from either vestibular end organs or the somatosensory system, or both. Two patients (13 and 14, refer supplementary material) demonstrated unique electrophysiological profiles, with a partial preservation of VEMP responses alongside sensory nerve abnormalities. Despite balance complaints and fatigue, their findings indicate a possible sensory polyneuropathy [58] or postganglionic lesions [50], highlighting further heterogeneity within the cohort. Overall, the constellation of auditory, vestibular, and somatosensory findings supports the notion that ANSD in this cohort may not represent an isolated auditory disorder but could be part of a broader multisystem neurological condition.

While the findings provide converging evidence for multisystem involvement in ANSD, the study has certain limitations. The absence of posturography limits the quantitative assessment of balance dysfunction. Additionally, replication in a larger sample is necessary to better delineate phenotypes and clarify underlying mechanisms.

## 5. Conclusions

This study provides clinical, vestibular, neurophysiological, and laboratory evidence that late-onset (acquired) ANSD extends beyond isolated auditory dysfunction and needs to be viewed as a multisystem neurological disorder. In addition to the characteristic audiological profile of ANSD, the patients demonstrated consistent balance and vestibular dysfunction, and objective evidence of somatosensory pathway involvement. The absent or reduced SNAPs, prolonged or absent lower limb SSEPs despite largely preserved motor nerve conduction, indicate selective involvement of the sensory afferent pathways. These abnormalities, together with evident vestibular deficits, provide a plausible neurophysiological basis for the balance difficulties observed in our cohort. Importantly, the findings suggest that a comprehensive neurological, vestibular, and somatosensory evaluation should be considered during the diagnostic work-up of individuals with late-onset ANSD.

## Use of AI tools declaration

The authors declare they have not used Artificial Intelligence (AI) tools in the creation of this article.


