# Supplementary material for: Subclinical neurological and balance abnormalities in late-onset auditory neuropathy spectrum disorder (ANSD) as a part of multisystem neurological presentation
Source: AIMS Neurosci. 2026 Jul 23;13(3):415–30. doi: 10.3934/Neuroscience.2026018 (PMC13658055; doi:10.3934/Neuroscience.2026018)
Supplement: Supplementary file 1 [file neurosci-13-03-018-s001.pdf]

Neuroscience

AIMS Neuroscience, 13(3): 415–430.
DOI: 10.3934/Neuroscience.2026018
Received: 19 January 2026
Revised: 16 April 2026
Accepted: 27 May 2026
Published: 23 July 2026

https://www.aimspress.com/journal/neuroscience

Research article

Subclinical neurological and balance abnormalities in late-onset auditory neuropathy spectrum disorder (ANSD) as a part of multisystem neurological presentation

Prashasti P Poovaiah[1,*], Atchayaram Nalini[2], Seena Vengalil[2], Suman Narayaswamy[3], Yamini Belur Keshavaprasad[3], Aravind Kumar Rajasekaran[3] and Pradeep Yuvraj[3]

[1] Department of Speech and Hearing, Father Muller College, Mangalore, Karnataka PO 575002, India
[2] Department of Neurology, National Institute of Mental Health and Neuroscience (NIMHANS), Bangalore, Karnataka PO 560029, India
[3] Department of Speech Pathology and Audiology, National Institute of Mental Health and Neuroscience (NIMHANS), Bangalore, Karnataka PO 560029, India

* **Correspondence:** Email: prashastipoovaiah@fathermuller.in; Tel: +917204920349.

Supplementary

Table 1. Demographics and clinical findings in subjects with auditory neuropathy spectrum disorder.

Patient Id/ age (Years)/ gender	1/23/F	2/17/F	3/25/M	4/21/M	5/16/F	6/15/F	7/16/M	8/15/F	9/15/F	10/20/M	11/22/M	12/17/F	13/20/F	14/22/M
Onset (age)	16 yrs	14yrs	15yrs	18yrs	13yrs	13yrs	14yrs	14yrs	13.5yrs	15yrs	15yrs	15yrs	17yrs	16yrs
Static (S)/ Progressive (P)	S	P	S	P	S	P	P	P	P	S	S	P	S	S
Hearing difficulty	+	+	+	+	+	+	+	+	+	+	+	+	+	+
Balance issues	Mild	yes	no	mild	mild	mild	no	no	yes	No	no	no	yes	mild
Fatigue	Mild	yes	no	yes	yes	yes	no	no	yes	Mild	mild	yes	yes	mild
Cranial Nerve assessment														
EOM	Full	Full	Full	Full	Full	Full	Full	Full	Full	Full	Full	Full	Full	Full
Ptosis	No	No	No	No	No	No	No	No	No	No	No	No	No	No
Diplopia	No	No	No	No	No	No	No	No	No	No	No	No	No	No
Nystagmus (gaze evoked)	No	No	No	No	No	No	No	No	No	No	No	No	No	No
Facial weakness	Mild	No	No	No	No	No	↓ sensation in left half of face	No	No	No	No	No	No	No
Tongue atrophy	no	+	No	No	No	No	No	No	+	No	No	No	Mild	No
Tongue fasciculation	+	+	Mild	+	-	-	Mild	+	+	+	+	+	+	+
Motor System														
Tone	Norm	Norm	Norm	Norm	Norm	Norm	Norm	Distal hypotonia in UL & LL	Norm	Norm	Norm	Norm	Norm	Norm

Power	5/5	5/5	5/5	5/5	5/5	5/5	5/5	5/5	5/5	5/5	5/5	5/5	5/5	5/5
Cerebellar system														
Finger-Nose	Norm	Norm	Norm	Norm	Norm	Norm	Norm	Norm	Norm	Norm	Norm	Norm	Norm	Norm
Knee-Heel	Norm	Norm	Norm	Norm	Norm	Norm	Norm	Norm	Norm	Norm	Norm	Norm	Norm	Norm
Disdiachokinesis	Norm	Norm	Norm	Norm	Norm	Norm	Norm	Norm	Norm	Norm	Norm	Norm	Norm	Norm
Sensory system														
Superficial sensation	Norm	Norm	Norm	Norm	Graded loss in LL	Norm	Norm	Norm	Norm	Norm	Norm	Norm	Norm	Norm
Touch, Pain, Temperature	Norm	norm	Norm	Norm	Norm	Norm	Norm	Norm	Norm	Norm	Norm	Norm	Norm	Norm
Joint-position statement	NP	NP	Norm	Impaired	Impaired in LL	Norm	Norm	Norm	↓in LL	Norm	NP	NP	Norm	Norm
Timed Vibration test	NP	NP	NP	↓in LL	NP	↓in LL	↓in LL	↓in LL	↓in LL	NP	↓in LL	Normal	NP	NP
Plantar response	↓↓	↓↓	↓↓	↓↓	↓↓	↓↓	↓↓	↓↓	↓↓	↓↓	↓↓	↓↓	↓↓	↓↓
Gait	Mild waddling	Mild waddling	Norm	Norm	Wide based	Norm	Norm	Norm	Norm	Norm	Norm	Norm	Mild waddling	Mild waddling
Romberg's test	+	+	+	+	+	+	+	+	+	+	+	+	+	+
Tandem Gait	+	+	+	+	+	+	+	+	+	+	+	+	+	+
Diagnosis	NDD	(?)ribofla-vinopathy	NDD	(?)BVVL	NDD	(?) Axonal neuropathy	(?)BVVL	(?)BVVL	(?)BVVL	(?)BVVL	(?)ribofla-vonopathy	(?)ribofla-vonopathy	NDD	NDD

Note: NDD: No definitive diagnosis, BVVL: Brown-Vialetto-Van Laere syndrome, UL: upper limbs, LL: lower limbs, '+': positive, '-': negative, Norm: Normal, ↓↓: reduced, NP: not performed.

Table 2. Electro-diagnostics and audiological findings in subjects with auditory neuropathy spectrum disorder (ANSD).

Patient ID/Age/Sex	Electro-diagnostics					Audiological findings									
	MCS		SCS		SSEP (latency)	PTA/SAL (dB HL)		Tympanometry/ Acoustic reflex		ABR	CM	OAE (DP)	cVEMP	oVEMP	vHIT
	Velocity	Amp.	Velocity	Amp.		Right	Left	Right	Left				Right/Left	Right/Left	Right/Left
1/23 yrs/F	Norm	Norm	SNAP absent in sural & SPN		N.P	51.6/ 30	30/ 15	'A'/ABS	'A'/ABS	ABS	PRES	PRES	ABS/ABS	ABS/ABS	Reduced gain/covert saccades
2/17 yrs/F	Norm	Norm	SNAP absent in sural & SPN		N.P	8.33/ 30	11.67/ 20	'A'/ABS	'A'/ABS	ABS	PRES	PRES	ABS/ABS	ABS/ABS	N.P
3/25 yrs/M	Norm	Reduced in CPN	Norm	Norm	Absent in LL	8.33/ 35	5/ 20	'A'/ABS	'A'/ABS	ABS	PRES	ABS/ABS	ABS/ABS	ABS/ABS	Reduced gain/covert saccades
4/21 yrs/M	Norm	Norm	Norm	Norm	Prolonged latencies in LL	13.3/ 20	15/ 20	'A'/ABS	'A'/ABS	ABS	PRES	ABS/ABS	ABS/ABS	ABS/ABS	Reduced gain/covert saccades
5/16 yrs/F	Norm	Norm	SNAP absent in Ulnar, radial, & sural		Absent in LL	36.6/ 40	30/ 30	'A'/ABS	'A'/ABS	ABS	PRES	PRES	ABS/ABS	ABS/ABS	N.P
6/15 yrs/F	Norm	Norm	Norm	Reduced in sural	Prolonged latencies in LL	36.6/ 35	30/ 25	'A'/ABS	'A'/ABS	ABS	PRES	ABS/ABS	ABS/ABS	ABS/ABS	Reduced gain/covert saccades
7/16 yrs/M	Norm	Norm	Norm	Norm	Prolonged latencies in LL	35.0/ 15	36.6/ 25	'A'/ABS	'A'/ABS	ABS	PRES	ABS/ABS	ABS/ABS	ABS/ABS	Reduced gain/covert saccades
8/15 yrs/F	Norm	Norm	SNAP absent in sural & SPN		Prolonged latencies in LL	76.6/ 50	51.6/ 35	'A'/ABS	'A'/ABS	ABS	PRES	PRES	ABS/ABS	ABS/ABS	N.P
9/15 yrs/F	Norm	Norm	Norm	Norm	N.P	41.6/ SRT-25	35/ SRT-25	'A'/ABS	'A'/ABS	ABS	PRES	ABS/ABS	ABS/ABS	ABS/ABS	N.P

10/19 yrs/M	Norm	Norm	SNAP absent in sural & SPN		Prolonged latencies in LL	71.6/ 65	63.3/ 35	‘A’/ABS	‘A’/ABS	ABS	PRES	PRES	ABS/ABS	ABS/ABS	Reduced gain/covert saccades
11/22 yrs/M	Norm	Norm	Norm	Norm	Norm	25/15	30/20	‘A’/ABS	‘A’/ABS	ABS	PRES	PRES	ABS/ABS	ABS/ABS	N.P
12/17 yrs/F	Norm	Norm	SNAP absent in sural & SPN		Absent in LL	66.6/80	55/65	‘A’/ABS	‘A’/ABS	ABS	PRES	PRES	ABS/ABS	ABS/ABS	Reduced gain/covert saccades
13/20 yrs/F	Norm	Norm	SNAP absent in sural & SPN		Absent in LL	65/40	66.6/15	‘A’/ABS	‘A’/ABS	ABS	PRES	PRES	ABS/ABS	PRES/PRES	Reduced gain/covert saccades
14/22 yrs/M	Norm	Norm	Norm		Norm	71.3/65	63.3/35	‘A’/ABS	‘A’/ABS	ABS	PRES	PRES	PRES/PRES	PRES/PRES	Reduced gain/covert saccades

Note: MCS: motor conduction study, SCS: sensory conduction study, SSEP: somatosensory evoked potential, CMAP: compound muscle action potential, SNAP: sensory nerve action potential, PTA: pure-tone average, SAL: speech awareness level, SRT: speech recognition threshold, SRS: speech recognition scores, SPIN: speech in noise test, ABR: auditory brainstem response, CM: cochlear microphonic, OAE(DP): oto-acoustic emission- distortion product, cVEMP: cervical vestibular evoked myogenic potential, oVEMP: ocular vestibular evoked myogenic potential, PRES: present, ABS: absent, Norm: normal, LL: lower limbs, SPN: superficial peroneal nerve, CPN: common peroneal nerve, dB HL: decibel hearing level, Amp.: amplitude, N.P: not performed.

AIMS Press © 2026 the Author(s), licensee AIMS Press. This is an open access article distributed under the terms of the Creative Commons Attribution License (https://creativecommons.org/licenses/by/4.0)
